# A survey of insecticide resistance-conferring mutations in multiple targets in *Anopheles sinensis* populations across Sichuan, China

**DOI:** 10.1186/s13071-021-04662-0

**Published:** 2021-03-20

**Authors:** Weiping Qian, Nian Liu, Yan Yang, Juan Liu, Jianhan He, Zuhua Chen, Mei Li, Xinghui Qiu

**Affiliations:** 1 Sichuan Center for Disease Control and Prevention, Chengdu, China; 2 State Key Laboratory of Integrated Management of Pest Insects and Rodents, Institute of Zoology, Chinese Academy of Sciences, Beijing, 100101 China; 3 Guangyuan Center for Disease Control and Prevention, Guangyuan, Sichuan Province China; 4 Neijiang Center for Disease Control and Prevention, Neijiang, Sichuan Province China; 5 Chengdu Center for Disease Control and Prevention, Chengdu, Sichuan Province China; 6 Panzhihua Center for Disease Control and Prevention, Panzhihua, Sichuan Province China

**Keywords:** *Anopheles sinensis*, Knockdown resistance (*kdr*), Acetylcholinesterase (AChE), Voltage-gated sodium channel (VGSC), Sichuan province of China

## Abstract

**Background:**

Sichuan province is located in the southwest of China, and was previously a malaria-endemic region. Although no indigenous malaria case has been reported since 2011, the number of imported cases is on the rise. Insecticide-based vector control has played a central role in the prevention of malaria epidemics. However, the efficacy of this strategy is gravely challenged by the development of insecticide resistance. Regular monitoring of insecticide resistance is essential to inform evidence-based vector control. Unfortunately, almost no information is currently available on the status of insecticide resistance and associated mechanisms in *Anopheles sinensis*, the dominant malaria vector in Sichuan. In this study, efforts were invested in detecting the presence and frequency of insecticide resistance-associated mutations in three genes that encode target proteins of several classes of commonly used insecticides.

**Methods:**

A total of 446 adults of *An. sinensis*, collected from 12 locations across Sichuan province of China, were inspected for resistance-conferring mutations in three genes that respectively encode acetylcholinesterase (AChE), voltage-gated sodium channel (VGSC), and GABA receptor (RDL) by DNA Sanger sequencing.

**Results:**

The G119S mutation in AChE was detected at high frequencies (0.40–0.73). The predominant *ace-1* genotype was GGC/AGC (119GS) heterozygotes. Diverse variations at codon 1014 were found in VGSC, leading to three different amino acid substitutions (L1014F/C/S). The 1014F was the predominant resistance allele and was distributed in all 12 populations at varying frequencies from 0.03 to 0.86. The A296S mutation in RDL was frequently present in Sichuan, with 296SS accounting for more than 80% of individuals in six of the 12 populations. Notably, in samples collected from Chengdu (DJY) and Deyang (DYMZ), almost 30% of individuals were found to be resistant homozygotes for all three targets.

**Conclusions:**

Resistance-related mutations in three target proteins of the four main classes of insecticides were prevalent in most populations. This survey reveals a worrisome situation of multiple resistance genotypes in Sichuan malaria vector. The data strengthen the need for regular monitoring of insecticide resistance and establishing a region-customized vector intervention strategy.

**Graphical abstract:**

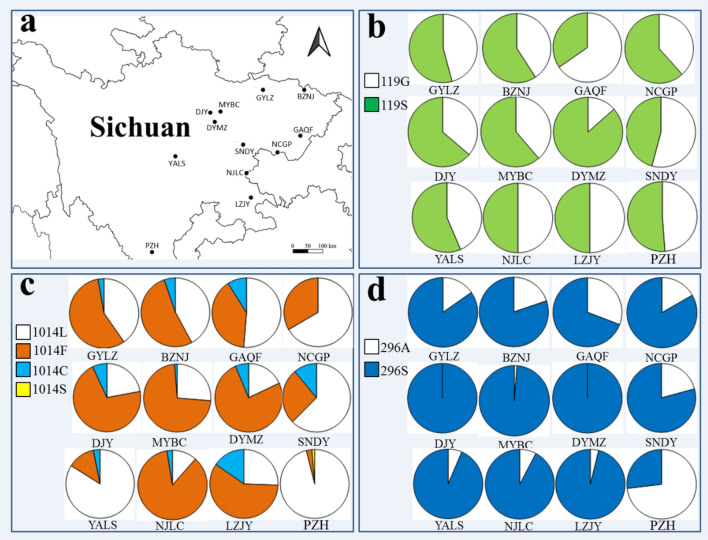

## Background

Malaria represents one of the most severe vector-borne diseases, posing a significant threat to global public health [1]. In 2018, an estimated 228 million cases of malaria occurred worldwide, with most malaria cases found in the WHO African Region (93%), followed by the WHO South-East Asia Region (3.4% of the cases). There were an estimated 405,000 deaths from malaria globally, notably children under the age of 5 years, accounting for 67% of all malaria deaths worldwide [2].

Vector control has played an essential role in the prevention of epidemics caused by indigenous cases and secondary infections induced by imported cases [3]. The control of malaria vectors has relied primarily on the use of various classes of insecticides via either indoor residual spraying (IRS) or treatment of bed nets. Organochlorines, organophosphates, carbamates, and pyrethroids are four groups of insecticides recommended by WHO for indoor residual spraying [4]. These insecticides have been heavily used in agriculture. The intensive use of these insecticides in mosquito-targeted control and in agriculture has led to widespread insecticide resistance in malaria vector species [5].

Sichuan province is located in the southwest of China, with a population of more than 80 million. Except for the northwest region with high altitude and cold weather, the natural environment in most parts of Sichuan is suitable for breeding of malaria vectors [6]. In fact, Sichuan was historically a malaria-endemic region. Thanks to the implementation of the National Malaria Control Programme launched in China in 1955, the malaria morbidity rate in Sichuan decreased from 87.4 per 10,000 people (580,771 patients in total) in 1954 to 0.017 per 10,000 people in 2012, and no indigenous malaria case has been reported since 2011 [6]. However, the number of imported cases is on the rise [6]. For example, a total of 290 imported malaria cases were reported in Sichuan province in 2015 [7], indicating that the risk of malaria resurgence remains.

*Anopheles sinensis* has become the dominant malaria vector in most regions of China including Sichuan [8, 9]. Given that resistance will diminish the effectiveness of current insecticide-based malaria vector-control interventions, regular insecticide resistance monitoring is essential to inform evidence-based vector control. Unfortunately, currently available information about the status of insecticide resistance in Sichuan *An. sinensis* is sparse. In this study, efforts were invested to reveal the molecular resistance status by detecting the presence and frequency of resistance alleles of three genes encoding targets of commonly used insecticides (i.e. acetylcholinesterase encoded by the *ace-1* gene, voltage-gated sodium channel encoded by the *vgsc* gene, and gamma-aminobutyric acid receptor encoded by the *rdl* gene) in *An. sinensis* populations collected from 12 sites across Sichuan province of China.

## Methods

*Anopheles sinensis* adults used in the study were caught around pigsties or cowsheds by light traps (wave length ~ 365 nm) between August and September 2018 from 12 locations across Sichuan province of China. In these regions, rice and vegetables are the main crops, and organophosphates (e.g. dichlorvos) and pyrethroids (e.g. cypermethrin, λ-cyhalothrin) are commonly used insecticides. Brief information about the 12 sample collection locations is listed in Table 1**.** Mosquitoes were trapped from 20:00 to 8:00 for 1 to 4 consecutive days at each location using 1–3 light traps, and were pooled for analysis. The specimens were morphologically identified and kept in 75 or 95% ethanol at 4 °C. Species identification was confirmed molecularly based on the nucleotide sequences of the second internal transcribed spacer (ITS2) region of the ribosomal DNA (rDNA) as described previously [10].

**Table 1:** Brief information of the sampling locations in Sichuan province of China

Sampling location	Code	Coordinates	Date
Bazhong Nanjiang	BZNJ	32°21′34″N, 106°76′02″E	August 2018
Chengdu Dujiangyan	DJY	30°96′26″N, 103°66′53″E	August 2018
Deyang Mianzhu	DYMZ	31°18′05″N, 104°15′53″E	August 2018
Guangan Qianfeng	GAQF	30°50′43″N, 106°67′76″E	August 2018
Guangyuan Lizhou	GYLZ	32°21′36″N, 105°49′60″E	August 2018
Luzhou Jiangyang	LZJY	28°47′75″N, 105°28′41″E	August 2018
Mianyang Beichuan	MYBC	31°37′97″N, 104°27′26″E	August 2018
Nanchong Gaoping	NCGP	30°17′49″N, 106°22′17″E	August 2018
Neijiang Longchang	NJLC	29°36′48″N, 105°19′53″E	September 2018
Panzhihua Miyi	PZH	27°0′10″N, 102°09′29″E	August 2018
Suining Daying	SNDY	30°33′03″N, 105°13′01″E	August 2018
Yaan Lushan	YALS	30°09′56″N, 102°56′29″E	August 2018

The genomic DNA (gDNA) of individual mosquitoes, excluding the abdomen, was isolated according to a previously described protocol [11]. Briefly, individual samples were placed in a tube with 0.5 ml of lysis buffer containing 100 mM Tris–Cl pH 8.0, 50 mM NaCl, and 10 mM EDTA, with 1% (w/v) SDS, 0.5 mM spermidine, 0.15 mM spermine, and 0.1 mg/ml (20 U/mg) proteinase K, and incubated at 60 °C for 20 min. After the addition of 75 µl of 8 M potassium acetate, the samples were mixed and set in an ice bath for 10 min and spun at 14,000×*g* for 5 min, after which the supernatant was transferred to a new tube. Then, 1 ml of absolute ethanol was added and the samples were kept at room temperature for 10 min. The samples were spun at 14,000×*g* for 10 min. Pellets were washed in 0.5 ml of 70% ethanol and spun at 14,000×*g* for 5 min. The pellets were dried and then re-suspended in H_2_O. The concentration of gDNA was determined using a NanoDrop 2000 spectrophotometer. gDNA samples were stored at −20 °C until use. Gene fragments containing codon 119 of *ace-1*, codon 1014 of *vgsc*, and codon 296 of *rdl* were amplified by PCR using the primers listed in Table 2. The PCR mixture (50 μl) consisted of 25 μl of 2× TIANGEN mix, 1 μl of each primer (5 μM), 1 μl of gDNA template, and ddH_2_O. The reactions were programmed as 95 °C for 2 min, 38 cycles of 94 °C for 30 s, 55–62 °C for 30 s (Table 2), 72 °C for 50 s, and an extension at 72 °C for 5 min.

**Table 2:** Brief information about PCR in this study

Name	Sequence (5′–-3′)	Annealing temperature (°C)	Amplicon size	References
ASACE-F	TAATGATCCGCTGGTGGTGA	60	~ 790 bp	ATLV01007054.1
ASACE-R	TACCGGAGAGTTGCTTCCTC			
ASKDR-F	TGCCACTCCGTGTGTTTAGA	55	~ 325 bp	Zhong et al. 2013
ASKDR-R	GAGCGATGATGATCCGAAAT			
ASRDL-F	AGTTTGTACGTTCGATGGGTTA	62	~ 476 bp	KE525297.1
ASRDL-R	GGCAACAGTAAGCTATGTCGA			

PCR products from individuals were visualized on agarose gels, and directly sequenced after purification using forward primers by TsingKe Company (Beijing, China). All sequencing data were checked manually. All confirmed DNA sequences were aligned by MUSCLE 3.8 [12], and nucleotide variations were documented. An independent chi-squared test was carried out to compare the overall difference in the allele frequency among *An. sinensis* populations by GraphPad Prism 9.0 (GraphPad Software, San Diego, CA, USA). Hardy–Weinberg equilibrium (HWE) was estimated using Genepop on the Web v.4.7.5 (https://genepop.curtin.edu.au/).

## Results

### Distribution and frequency of *ace-1* genotypes

**Fig. 1:**
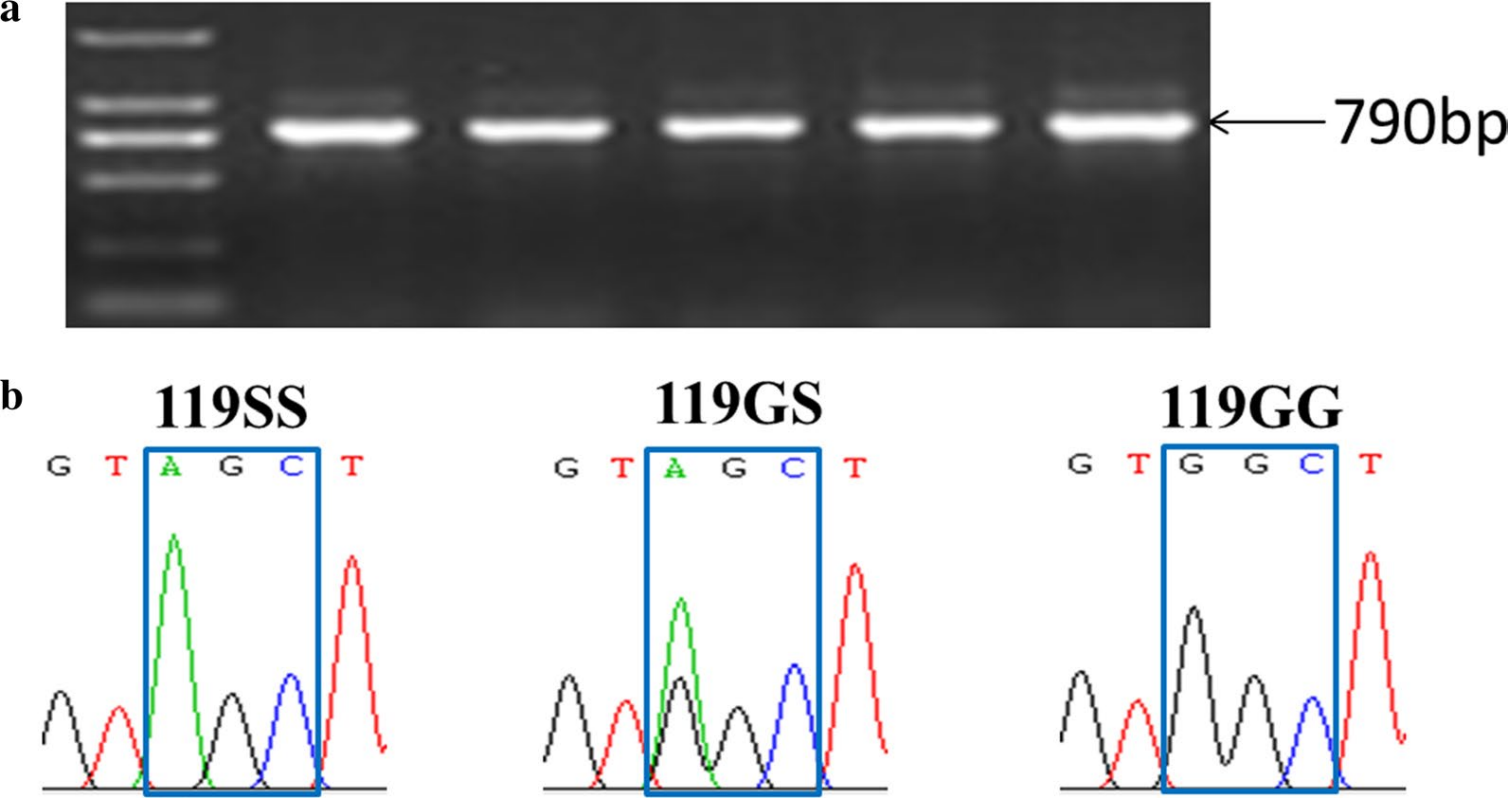
Example photo of amplicons on agarose gel (**a**), and chromatograms showing the genotypes encoding AChE-119 detected in this study (**b**)

A variation (GGC to AGC) that causes an amino acid substitution (G to S) was observed at the first nucleotide of codon 119 of the *ace-1* gene (Fig. 1). The G119S replacement in the AChE can confer resistance to organophosphorus (OP) and carbamate (CM) insecticides. All three possible genotypes (119GG, 119GS, 119SS) were detected (Fig. 1; Table 3). The predominant genotype was the 119GS heterozygote: over 50% of individuals were 119GS in 11 of the 12 tested populations. In contrast, the susceptible homozygotes (119GG) were relatively rare, with frequencies ranging from 0 to 0.31. Appreciable frequencies (0.08–0.46) of resistant homozygotes were detected (Table 3).

**Table 3:** Frequency of individual *ace-1* genotypes and alleles

Populations	*N*	Genotypes			HWE test (*p* value)		Alleles*	
		GG	GS	SS	Probability test	Heterozygote excess	119G	119S
BZNJ	38	0.11	0.60	0.29	0.18	0.12	0.41	0.59
DJY**	40	0.02	0.68	0.30	0.01	0.00	0.36	0.64
DYMZ**	39	0	0.54	0.46	0.04	0.02	0.27	0.73
GAQF	39	0.31	0.59	0.10	0.18	0.10	0.60	0.40
GYLZ	36	0.11	0.64	0.25	0.10	0.08	0.43	0.57
LZJY	39	0.23	0.54	0.23	0.75	0.47	0.50	0.50
MYBC**	40	0.05	0.68	0.27	0.02	0.01	0.39	0.61
NCGP	30	0.17	0.43	0.40	0.70	0.83	0.38	0.62
NJLC**	39	0.08	0.84	0.08	0.00	0.00	0.50	0.50
PZH	39	0.15	0.67	0.18	0.06	0.04	0.49	0.51
SNDY**	36	0.19	0.69	0.11	0.04	0.02	0.54	0.46
YALS**	31	0.06	0.74	0.19	0.01	0.01	0.44	0.56
Average		0.12	0.64	0.24	–	–	0.44	0.56

*N* = total number of individuals detected in a specific location. The abbreviations for the sampling locations are shown in Table 1

*Chi-square test: *χ*^2^ = 27.51, df = 11, *p* = 0.0038

**Population not in conformity to Hardy–Weinberg equilibrium at *p* value ≤ 0.05

### Distribution and frequency of *kdr* genotypes

**Fig. 2:**
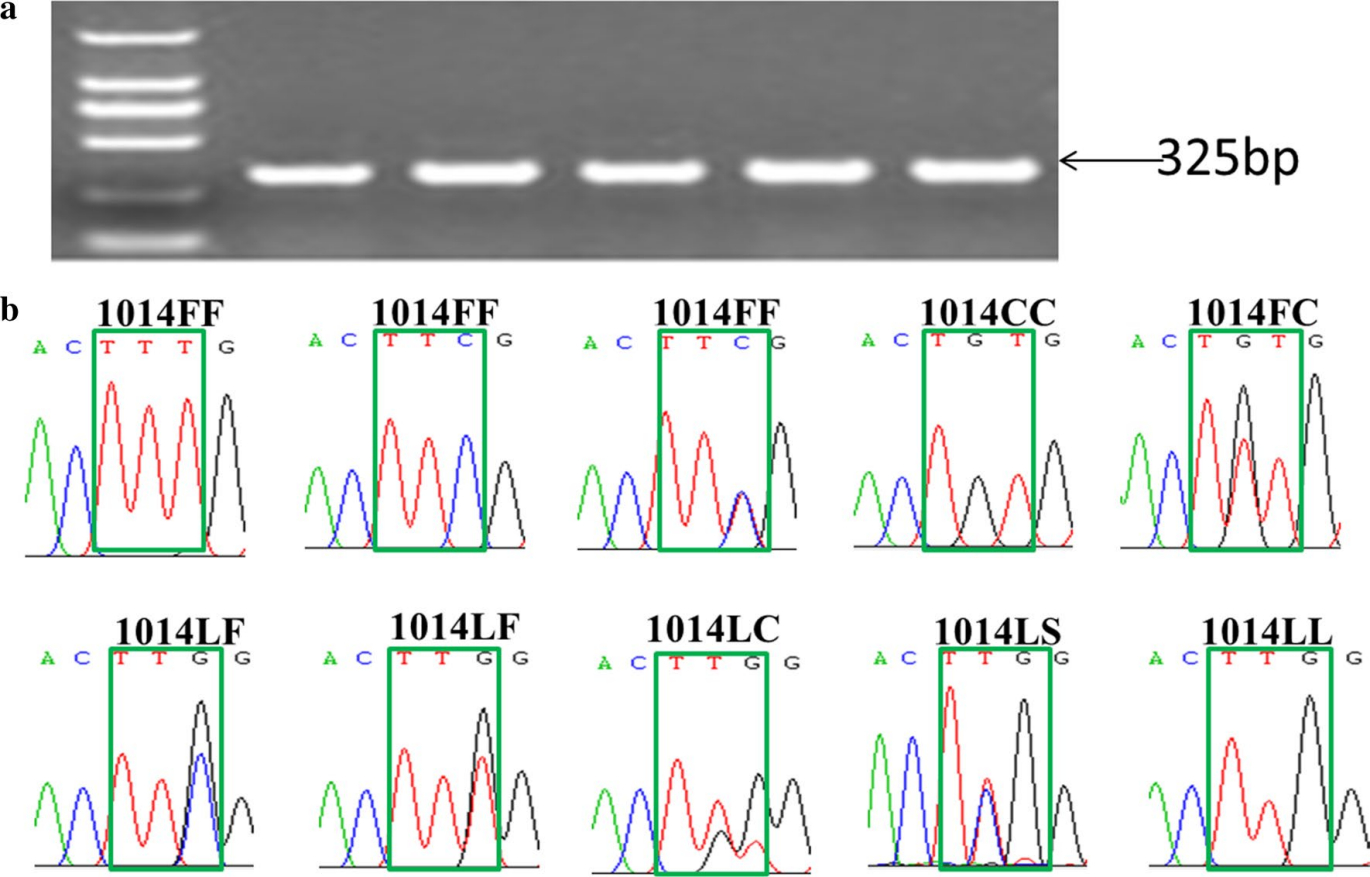
Example photo of amplicons on agarose gel (**a**) and chromatograms showing the genotypes encoding VGSC-1014 detected in this study (**b**)

Variations were detected at the second (T to G or C) and third nucleotide (G to T or C) of codon 1014 of the *vgsc* gene, leading to three amino acid substitutions at position 1014 (L to F/C/S) (Fig. 2). Ten different genotypes were identified in total, and 1014F was observed to be encoded by either TTC or TTT (Fig. 2). Based on the amino acid at position 1014, three to five different *vgsc* genotypes were distributed in a specific population (Table 4). Genotypes LL, FF, LF, and LC were widely distributed, while LS and CC were found only in PZH and LZJY, at very low frequency, respectively. LF and FF were the predominant genotypes in most locations.

**Table 4:** Frequency of *vgsc* individual genotypes and alleles

	N	Genotypes							HWE test		Alleles^+^			
		LL	LF*	LC	LS	FF**	FC	CC	Probability test	Heterozygote excess	1014L	1014F	1014C	1014S
BZNJ	38	0.13	0.53	0.05	0	0.24	0.05	0	0.62	0.17	0.42	0.53	0.05	0
DJY	40	0.03	0.28	0.03	0	0.55	0.13	0	1.00	0.58	0.18	0.75	0.07	0
DYMZ	39	0.03	0.28	0.03	0	0.56	0.10	0	1.00	0.49	0.18	0.76	0.06	0
GAQF	39	0.31	0.31	0.10	0	0.21	0.08	0	0.51	0.77	0.51	0.40	0.09	0
GYLZ	36	0.11	0.53	0.06	0	0.31	0	0	0.16	0.20	0.40	0.57	0.03	0
LZJY	39	0	0.44	0.08	0	0.28	0.18	0.03	0.13	0.06	0.26	0.59	0.15	0
MYBC	40	0.03	0.45	0.03	0	0.50	0	0	0.18	0.17	0.26	0.73	0.01	0
NCGP	30	0.40	0.53	0	0	0.07	0	0	0.43	0.28	0.67	0.33	0.00	0
NJLC	39	0.03	0.15	0.03	0	0.77	0.03	0	0.15	0.93	0.11	0.86	0.03	0
PZH	39	0.92	0.05	0.00	0.03	0	0.00	0	1.00	0.96	0.96	0.03	0.00	0.01
SNDY	36	0.33	0.42	0.17	0	0.05	0.03	0	0.65	0.17	0.63	0.28	0.09	0
YALS	31	0.68	0.19	0.03	0	0.10	0	0	0.09	0.99	0.79	0.19	0.02	0
Average	-	0.25	0.35	0.05	–	0.30	0.05	–	–	–	0.45	0.50	0.05	–

*N* = total number of individuals detected in a specific location. The abbreviations for the sampling locations are shown in Table 1

*Including TTG/C and TTC/T; **including TTT, TTC, and TTC/T

^+^Chi-square test:* χ*^2^ = 286.4, df = 33, *p* < 0.0001

### Distribution and frequency of *rdl* genotypes

**Fig. 3:**
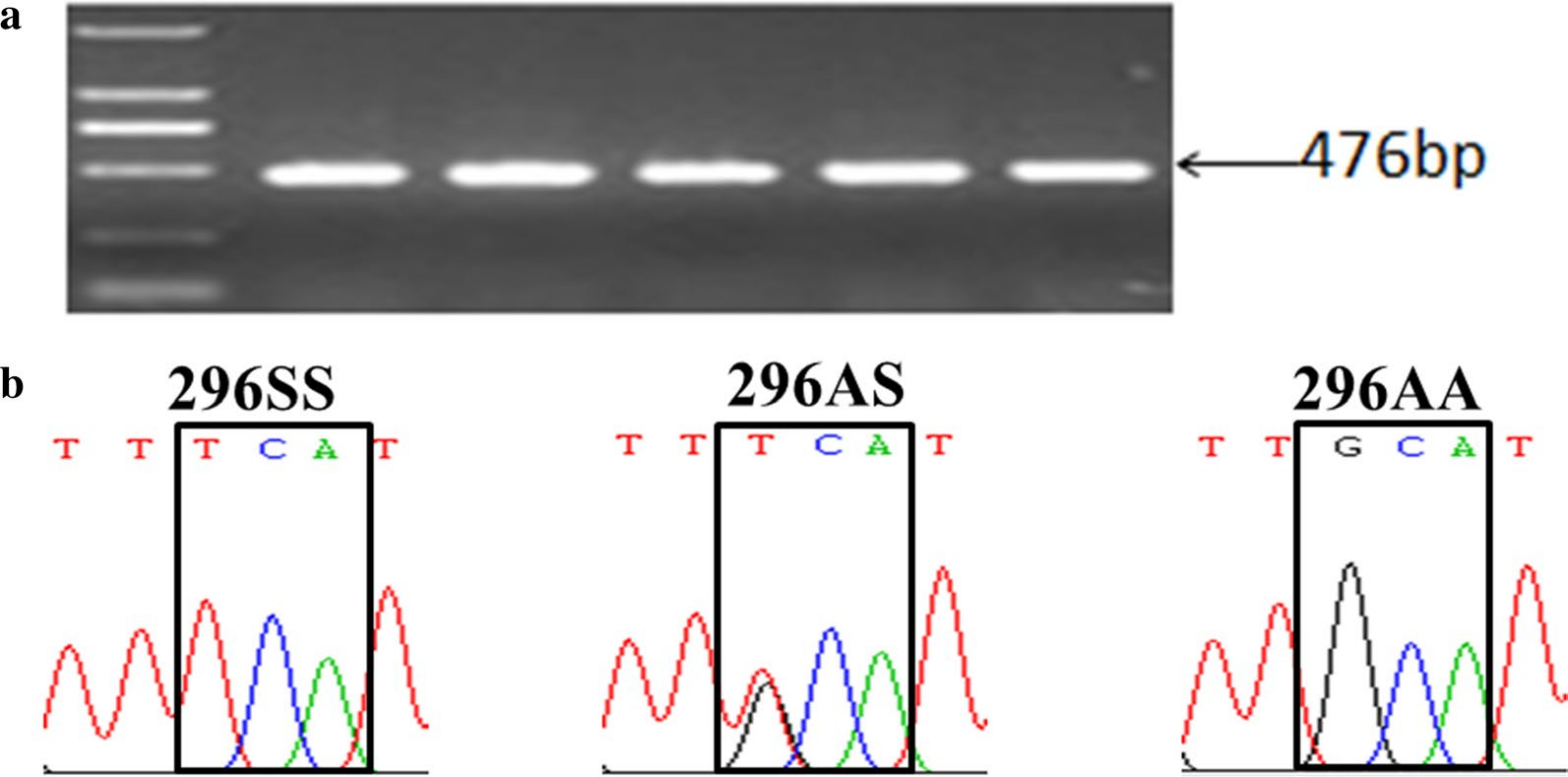
Example photo of amplicons on agarose gel (**a**) and chromatograms showing the genotypes encoding RDL-296 detected in this study (**b**)

DNA sequencing identified a non-silent mutation at the first nucleotide of codon 296 of the *rdl* gene, leading to a deduced amino acid substitution of A (GCA) to S (TCA) (Fig. 3). Three different genotypes (296SS, 296AS, and 296AA) were detected in our specimens (Table 5). With the exception of PZH, the frequency of mutant homozygotes was high, with 296SS accounting for more than 80% of individuals in six of the 12 populations. Notably, the wild 296AA homozygote was not detectable in eight of the 12 populations, while 100% of individuals were resistant 296SS homozygotes in DJY and DYMZ.

**Table 5:** Frequency of *rdl* individual genotypes and alleles

Locations	*N*	Genotypes			HWE test		Alleles*	
		AA	AS	SS	Probability test	Heterozygote excess	296A	296S
BZNJ	38	0	0.39	0.61	0.31	0.18	0.20	0.80
DJY	40	0	0	1.00	–	–	0	1.00
DYMZ	39	0	0	1.00	–	–	0	1.00
GAQF	39	0.18	0.26	0.56	0.02	1.00	0.31	0.69
GYLZ	36	0	0.31	0.69	0.57	0.40	0.15	0.85
LZJY	39	0	0.08	0.92	1.00	0.96	0.04	0.96
MYBC	40	0	0.03	0.97	–	–	0.03	0.97
NCGP	30	0	0.33	0.67	0.56	0.41	0.17	0.83
NJLC	39	0	0.15	0.85	1.00	0.81	0.08	0.92
PZH	39	0.56	0.33	0.10	0.42	0.92	0.78	0.22
SNDY	36	0.08	0.25	0.67	0.15	0.98	0.21	0.79
YALS	31	0.03	0.06	0.90	0.10	1.00	0.06	0.94
Average		0.07	0.18	0.75	–	–	0.17	0.83

*N* = total number of individuals detected in a specific location. The abbreviations for the sampling locations are shown in Table 1

*Chi-square test:* χ*^2^ = 283.3, df = 11, *p* < 0.0001

### Distribution and frequency of triple-target genotype combinations

From the 446 individuals, 34 triple-target genotype combinations were documented (Table 6). Among these, C18 (119GS + 1014LF + 296SS) and C23 (119GS + 1014FF + 296SS) were the most widely distributed combinations. Moreover, the three-target resistance homozygous genotype (C33) was also widely distributed. Most notably, almost 30% of individuals were found to be resistant homozygotes (C33 and C34) for all three targets in DJY and DYMZ.

**Table 6:** Distribution and frequency of triple-target genotype combinations

	ACE119 VGSC1014 RDL296	*N*	BZNJ	DJY	DYMZ	GAQF	GYLZ	LZJY	MYBC	NCGP	NJLC	PZH	SNDY	YALS
C1	GG LL AA	9				5						3	1	
C2	GG LL AS	7				3				1		2	1	
C3	GG LL SS	6								4			1	1
C4	GG LF AA	1										1		
C5	GG LF AS	4	1			1		1						1
C6	GG LF SS	11	2	1		2	2	2					2	
C7	GG LC AS	1											1	
C8	GG LC SS	2				1		1						
C9	GG FF AS	1						1						
C10	GG FF SS	10	1				2	2	2		3			
C11	GG FC SS	2						1					1	
C12	GG CC SS	1						1						
C13	GS LL AA	17										15	2	
C14	GS LL AS	20	2			1	2			3		9	3	
C15	GS LL SS	27	1		1	2	1			1	1	2	3	15
C16	GS LF AA	1				1								
C17	GS LF AS	24	6			3	7	1		2	1		3	1
C18	GS LF SS	78	4	9	5	5	8	0	15	6	5		8	3
C19	GS LC AS	3				1	1						1	
C20	GS LC SS	14	3	1	1	2		2			1		4	1
C21	GS LS AS	1										1		
C22	GS FF AS	7				1			1		5			
C23	GS FF SS	80	6	14	12	5	5	4	11	1	19		1	2
C24	GS FC AA	1				1								1
C25	GS FC SS	14	2	3	2	1		4			1			
C26	SS LL AA	3										3		
C27	SS LL AS	4	2							2				
C28	SS LL SS	12				1	1		1	1		2	1	5
C29	SS LF AS	5	3							1		1		
C30	SS LF SS	31	4	1	6	1	2	3	4	7			2	1
C31	SS LC SS	3				1	1		1					
C32	SS FF AS	3	1				1			1				
C33	SS FF SS	37	1	9	10	1	3	4	5		3		1	
C34	SS FC SS	6		2	2			2						
	Total	446	38	40	39	39	36	39	40	30	39	39	36	31

*N* = number of individuals. The abbreviations for the sampling locations are shown in Table 1

Resistance-related mutations in multiple insecticide targets were prevalent in most *Anopheles sinensis* populations in Sichuan, China

### Distribution and frequency of resistance alleles

**Fig. 4:**
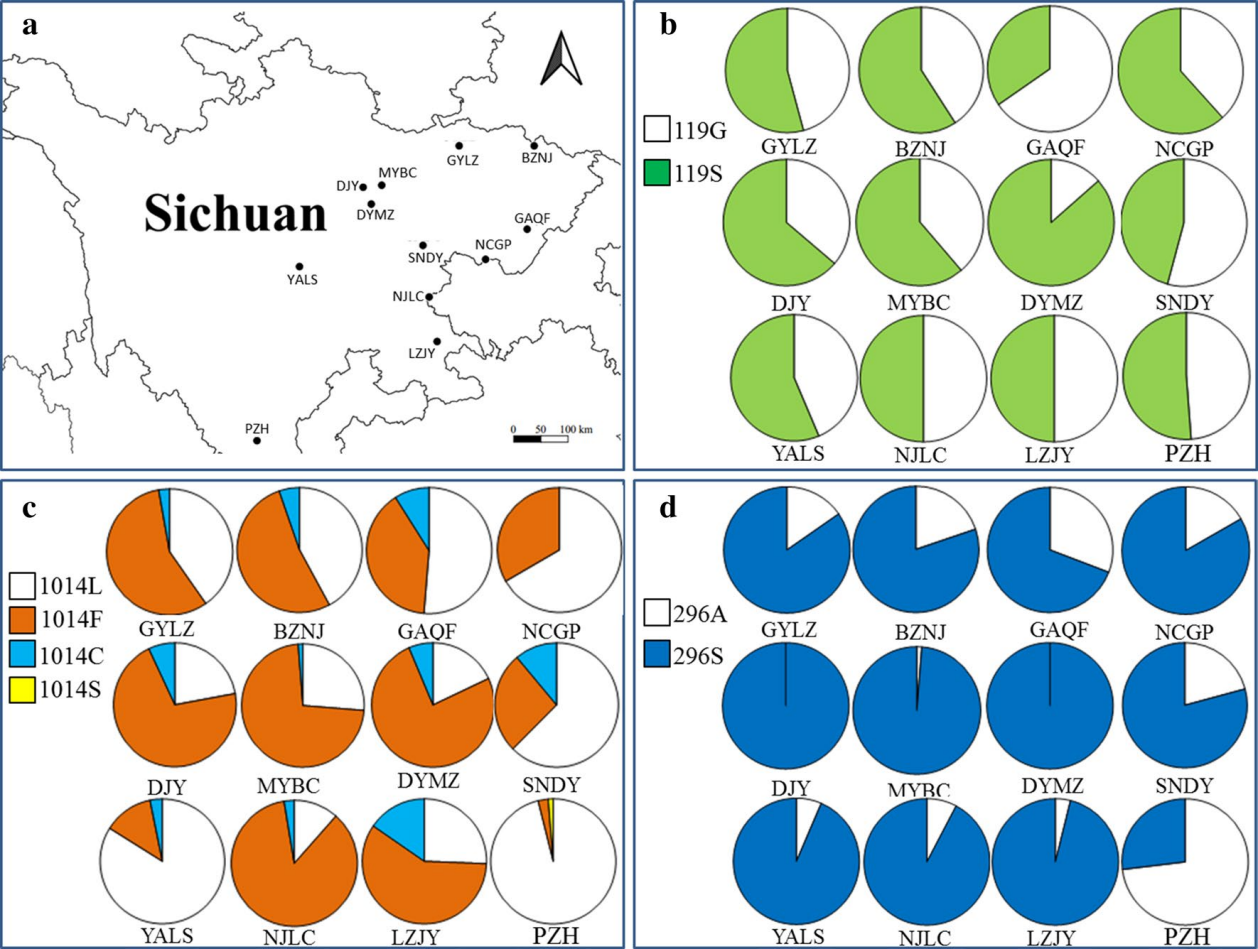
Distribution and frequency of alleles in *Anopheles sinensis* populations in Sichuan. **a** Map showing the sampling sites. **b** AChE-119; C, VGSC-1014; D, RDL-296

The frequency of resistance alleles is summarized in Fig. 4. AChE1-119S was found at a frequency ranging between 0.40 and 0.73 (Table 3). VGSC-1014F was the predominant resistance allele in each location, and was distributed in all 12 populations, with frequency varying from 0.03 to 0.86. VGSC-1014C was present in ten populations, with frequency of 0.02–0.15, while VGSC-1014S was observed only in PZH, with a frequency of 0.013 (Table 4). High frequencies of RDL-296S (0.69–1.00) were detected in 11 locations, the exception being PZH (Table 5). Chi-square tests indicated that the insecticide resistance-related mutations were heterogeneously distributed in the 12 populations (Tables 3, 4, 5).

## Discussion

The control of disease-borne insects has heavily relied on the use of insecticides. The evolution of insecticide resistance worldwide has been well recognized as a major obstacle in effective vector control [1]. Implementation of vector-control interventions should take into account the resistance situation of local disease vectors. However, there have been almost no published data to inform the status and underlying genetic mechanisms of insecticide resistance in the malaria vector *An. sinensis* in Sichuan. The current work represents the first extensive survey of target-site mutations in *An. sinensis* populations across Sichuan.

Acetylcholinesterase is the primary molecular target of organophosphates (OP) and carbamates (CM). The G119S replacement in AChE is associated with OP and CM resistance in several important mosquito species [13–17]. In Sichuan, this conservative mutation was detected in all *An. sinensis* populations (Table 2 and Fig. 4). This result is in keeping with observations in published literature indicating that the G119S mutation is widely distributed in *An. sinensis* in Asia [10, 18–21]. The high frequency (0.40–0.73, with an average of 0.56) of the resistant 119S allele strongly indicates the occurrence of appreciable resistance to OP and CM in these regions.

VGSC is the major target for pyrethroids and dichlorodiphenyltrichloroethane (DDT) [21]. Many studies have demonstrated that mutations at codon 1014 of the *vgsc* gene are able to confer resistance to both pyrethroids and DDT in many arthropod species including anophelines [22, 23]. In *An. sinensis*, significant positive correlations have been found between *kdr* allele frequency and bioassay-based resistance phenotype, and three different mutations of VGSC at position 1014 (1014F/C/S) have been documented [20, 21, 24–28]. We found that all three mutations were present in Sichuan. In contrast to the situation with *An. sinensis* in Guangxi, China, where relatively higher frequencies of 1014C or 1014S than 1014F were observed [21, 27], 1014F is the predominant resistance allele in Sichuan (Table 4; Fig. 4).

The insect gamma-aminobutyric acid (GABA) receptor RDL subunit encoded by the *rdl* (resistance to dieldrin) gene plays a central role in neuronal signaling and is involved in various processes [29]. RDL has been the primary target for insecticides of various chemical structures including cyclodienes and fipronil [29], and a potential secondary target for neonicotinoids and pyrethroids [30]. In this study, the A296S mutation was identified and found to be widely distributed in *An. sinensis* populations across Sichuan (Table 5; Fig. 4). These data would predict a risk of resistance to the old cyclodienes and relatively new phenylpyrazoles in Sichuan populations of *An. sinensis*.

For *vgsc* 1014 and *rdl* 327 loci, genotype frequencies were detected in conformity to HWE (Tables 4 and 5). However, there was a significant departure from the HWE at the *ace-1* 119 locus in six of the 12 populations (Table 3). Significant heterozygote excess may suggest that there is a heterologous duplication in *ace-1* in these populations, although the possibility of selection for heterozygotes in the field cannot be excluded. Duplication of the *ace-1* gene has been reported in several mosquito species including *Anopheles* and *Culex* [31–34], but not in *An. sinensis*, to the best of our knowledge. Previous studies have demonstrated that permanent *ace-1* heterozygotes exhibit resistance to both OP and CM, and a reduction of fitness costs [32, 33], whether heterologous duplication is present in *An. sinensis ace-1* deserves further investigation.

Taken together, the results show that several well-known genetic mutations associated with insecticide resistance in *An. sinensis* are widely distributed with high frequency in Sichuan. This situation may be explained in part by the application of a large amount of insecticides immediately after the 2008 Sichuan earthquake and/or insecticide-based vector-control campaigns for building “healthy cities” in recent decades. Moreover, this survey reveals the presence of individuals harboring mutations in more than one insecticidal target (Table 6). Even worse, in DJY and DYMZ, about 30% of individuals were resistant homozygotes for all three targets (Table 6).

## Conclusions

In this survey, we found the occurrence of resistance-related mutations in multiple targets of the four main classes of insecticides. Notably, these target site mutations were present at high frequencies in most *An. sinensis* populations. Geographical heterogeneities of allele frequency among different locations were significant. These findings emphasize the need to establish a location-customized resistance management strategy before implementing insecticide-based malaria control programmes.

## Data Availability

All datasets are presented in this published article.

## Abbreviations

AChE: Acetylcholinesterase
CM: Carbamate insecticides
GABA: Gamma-aminobutyric acid
*kdr*: Knockdown resistance
OC: Organochlorine insecticides
OP: Organophosphorus insecticides
PCR: Polymerase chain reaction
PY: Pyrethroid insecticides
VGSC: Voltage-gated sodium channel
RDL: Resistance to dieldrin
